# Lymphangiogenesis in renal fibrosis arises from macrophages via VEGF-C/VEGFR3-dependent autophagy and polarization

**DOI:** 10.1038/s41419-020-03385-x

**Published:** 2021-01-21

**Authors:** Ying Zhang, Conghui Zhang, Lixi Li, Xinjun Liang, Peng Cheng, Qing Li, Xiaoyan Chang, Kun Wang, Shuai Huang, Yueqiang Li, Yanyan Liu, Gang Xu

**Affiliations:** 1 Department of Nephrology, Division of Internal Medicine, Tongji Hospital, Tongji Medical College, Huazhong University of Science and Technology, Wuhan, Hubei China; 2 Department of Medical Oncology, Hubei Cancer Hospital, Tongji Medical College, Huazhong University of Science and Technology, Wuhan, Hubei China; 3 Department of Orthopaedics, Tongji Hospital, Tongji Medical College, Huazhong University of Science and Technology, Wuhan, Hubei China; 4 Daping Hospital, Army Medical University (The Third Military Medical University), Chongqing, China

**Keywords:** Lymphangiogenesis, End-stage renal disease

## Abstract

Inflammation plays a crucial role in the occurrence and development of renal fibrosis, which ultimately results in end-stage renal disease (ESRD). There is new focus on lymphangiogenesis in the field of inflammation. Recent studies have revealed the association between lymphangiogenesis and renal fibrosis, but the source of lymphatic endothelial cells (LECs) is not clear. It has also been reported that macrophages are involved in lymphangiogenesis through direct and indirect mechanisms in other tissues. We hypothesized that there was a close relationship between macrophages and lymphatic endothelial progenitor cells in renal fibrosis. In this study, we demonstrated that lymphangiogenesis occurred in a renal fibrosis model and was positively correlated with the degree of fibrosis and macrophage infiltration. Compared to resting (M0) macrophages and alternatively activated (M2) macrophages, classically activated (M1) macrophages predominantly transdifferentiated into LECs in vivo and in vitro. VEGF-C further increased M1 macrophage polarization and transdifferentiation into LECs by activating VEGFR3. It was suggested that VEGF-C/VEGFR3 pathway activation downregulated macrophage autophagy and subsequently regulated macrophage phenotype. The induction of autophagy in macrophages by rapamycin decreased M1 macrophage polarization and differentiation into LECs. These results suggested that M1 macrophages promoted lymphangiogenesis and contributed to newly formed lymphatic vessels in the renal fibrosis microenvironment, and VEGF-C/VEGFR3 signaling promoted macrophage M1 polarization by suppressing macrophage autophagy and then increased the transdifferentiation of M1 macrophages into LECs.

## Introduction

Recently, chronic kidney disease (CKD) has become a global public health concern. As the disease progresses, the glomerular filtration rate decreases and patient life quality progressively declines, eventually resulting in end-stage renal disease (ESRD), which causes heavy social and personal burdens; thus, CKD prevention is currently the focus of clinical attention. Studies have shown that renal fibrosis is the final outcome of CKD progression, manifesting as extracellular matrix deposition and progressive glomerular and tubular fibrosis and leading to a gradual loss of renal function^1^. Therefore, it is of great importance to clarify the mechanism by which renal fibrosis occurs and develops to prevent and treat CKD.

Many factors, such as inflammation, endothelial/epithelial cell transdifferentiation into mesenchymal cells, oxidative stress, and myofibroblast recruitment, have been found to play important roles in the development of renal fibrosis. Among these factors, immune factors also respond to nonimmune stimuli and are therefore considered to be the core of renal fibrosis^2^. An increasing number of studies have shown that lymphatic vessels not only are passive drainage channels but also actively participate in a variety of biological effects. These vessels are essential for immune regulation, immune surveillance, and inflammatory responses and are also new targets for immune-related disease intervention^3^. Reports have shown that lymphangiogenesis, an immune-related factor, develops in CKD patients with different pathologies^4^, but the specific role and mechanism are not clear^5^. Therefore, it is of great importance to clarify the mechanism by which lymphangiogenesis occurs in renal fibrosis to prevent and treat CKD.

Studies have shown that many factors and cells are involved in lymphangiogenesis, the most classic of which is vascular endothelial growth factor-C (VEGF-C), which binds to the receptor VEGFR-3 and is crucial for the proliferation, migration, and survival of lymphatic vascular endothelial cells^6,7^. In a variety of tissue inflammatory microenvironments, lymphatic endothelial cells (LECs) can effectively form new lymphatic vessels according to changes in the microenvironment^3^. However, Kerjaschki et al. found that the Y chromosome could be detected in newly formed LECs in the biopsy tissue of male transplanted kidney from a female donor, suggesting that renal lymphangiogenesis comes from not only innate cell proliferation but also progenitor cells from the blood circulation^8^. As early as 2005, it was found that new lymphatics colocalized with macrophages during corneal injury repair^9^. Myeloid-derived LECs progenitors are similarly recruited to tumors and integrate into pre-existing lymphatic systems^10^. Whether macrophages can become such progenitor cells in renal fibrosis is unknown. Therefore, this study was designed to investigate the relationship between macrophage infiltration and lymphangiogenesis in renal fibrosis, whether macrophages in renal fibrosis can transdifferentiate into LECs, and what role VEGF-C/VEGFR3, the most classic lymphangiogenic growth factor, plays in this process. Autophagy has been identified as a factor that modulates macrophage polarization^11^, and so macrophage autophagy was also examined in this study.

## Results

### The relationship between renal fibrosis, macrophage infiltration, and lymphangiogenesis in the mouse model

**Fig. 1:**
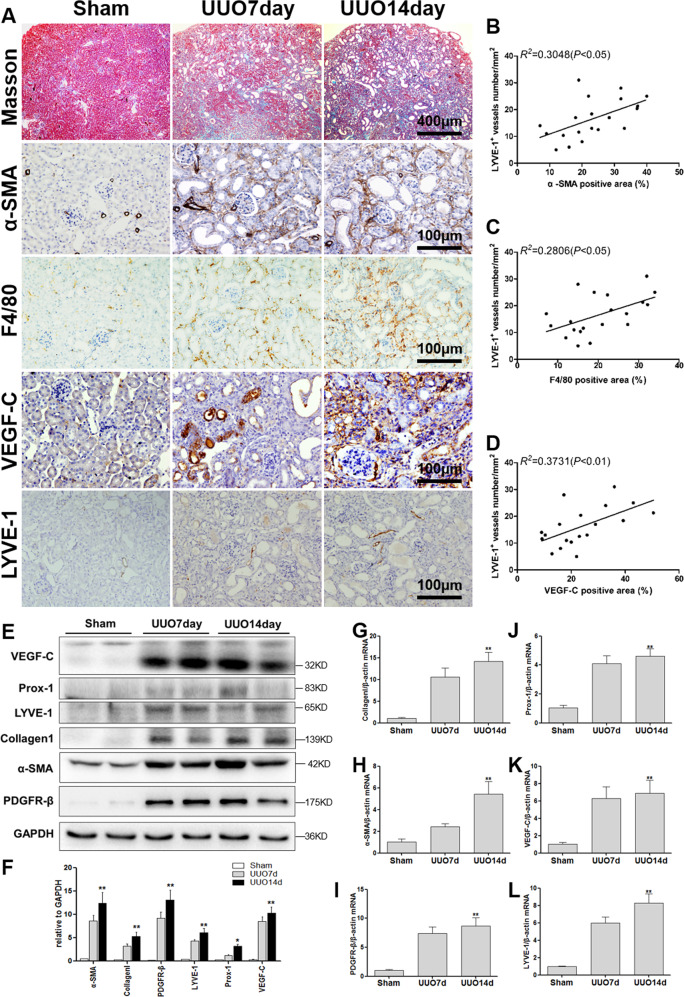
Renal fibrosis, lymphangiogenesis, macrophage infiltration, and VEGF-C expression in UUO mice. **A** Kidney tissue from sham-operated (Sham) or unilateral ureteral obstruction (UUO) mice was tested by Masson staining (100×) and immunohistochemical analysis of α-SMA, F4/80, VEGF-C, and LYVE-1 (400×). There was a positive correlation between the number of LYVE-1^+^ vessels and the positive area (%) of α-SMA (**B**), F4/80 (**C**), and VEGF-C (**D**). **E** Protein expression of fibrosis markers (α-SMA, collagen 1, and PDGFR-β) and lymphangiogenesis markers (LYVE-1, Prox-1, and VEGF-C) in Sham and UUO mouse kidneys were measured by western blotting. **F** Quantitative analysis of the results in (**E**) was conducted to show the protein expression of fibrosis markers (α-SMA, collagen 1, and PDGFR-β) and lymphangiogenesis markers (LYVE-1, Prox-1, and VEGF-C) in Sham and UUO mouse kidneys. *p < 0.05, **p < 0.01 versus the Sham group. **G**–**L** Relative mRNA expression of Collagen 1 (**G**), α-SMA (**H**), PDGFR-β (**I**), Prox-1 (**J**), VEGF-C (**K**), and LYVE-1 (**L**) in Sham and UUO mouse kidneys was measured by real-time PCR; *p < 0.05, **p < 0.01 versus the Sham group. *N* = 6/group.

To explore the relationship between macrophage infiltration and lymphangiogenesis in renal fibrosis, UUO, and ADR mice were used. Kidney tissue and sections were obtained for Masson staining, IHC staining, western blotting, and real-time PCR. As the obstruction time increased (7 and 14 days), tubular dilation, degeneration, and atrophy gradually became severe, so it was true for renal fibrosis (Masson, α-SMA), macrophage infiltration (F4/80), and lymphangiogenesis (LYVE-1). VEGF-C is known to be an important growth factor associated with lymphangiogenesis, and its expression was also gradually upregulated (Fig. 1A). Then, semi-quantitative analysis and correlation analysis of the IHC staining results were conducted, and the density of LYVE-1^+^ lymphatic vessels correlated linearly with the expression of α-SMA (*r*^2^ = 0.6179, *P* < 0.01) (Fig. 1B), F4/80 (*r*^2^ = 0.5094, *P* < 0.01) (Fig. 1C) and VEGF-C (*r*^2^ = 0.4471, *P* < 0.01) (Fig. 1D). Western blotting (Fig. 1E, F) and real-time PCR (Fig. 1G-L) results also showed that lymphangiogenesis (VEGF-C, LYVE-1, Prox-1) was upregulated as fibrosis became severe (Collagen1, α-SMA, PDGFR-β). There was also a similar effect in the ADR (Fig. S1). These results demonstrated that lymphangiogenesis paralleled renal fibrosis, macrophage infiltration, and VEGF-C expression.

### Lymphangiogenesis is downregulated in UUO after macrophage depletion

**Fig. 2:**
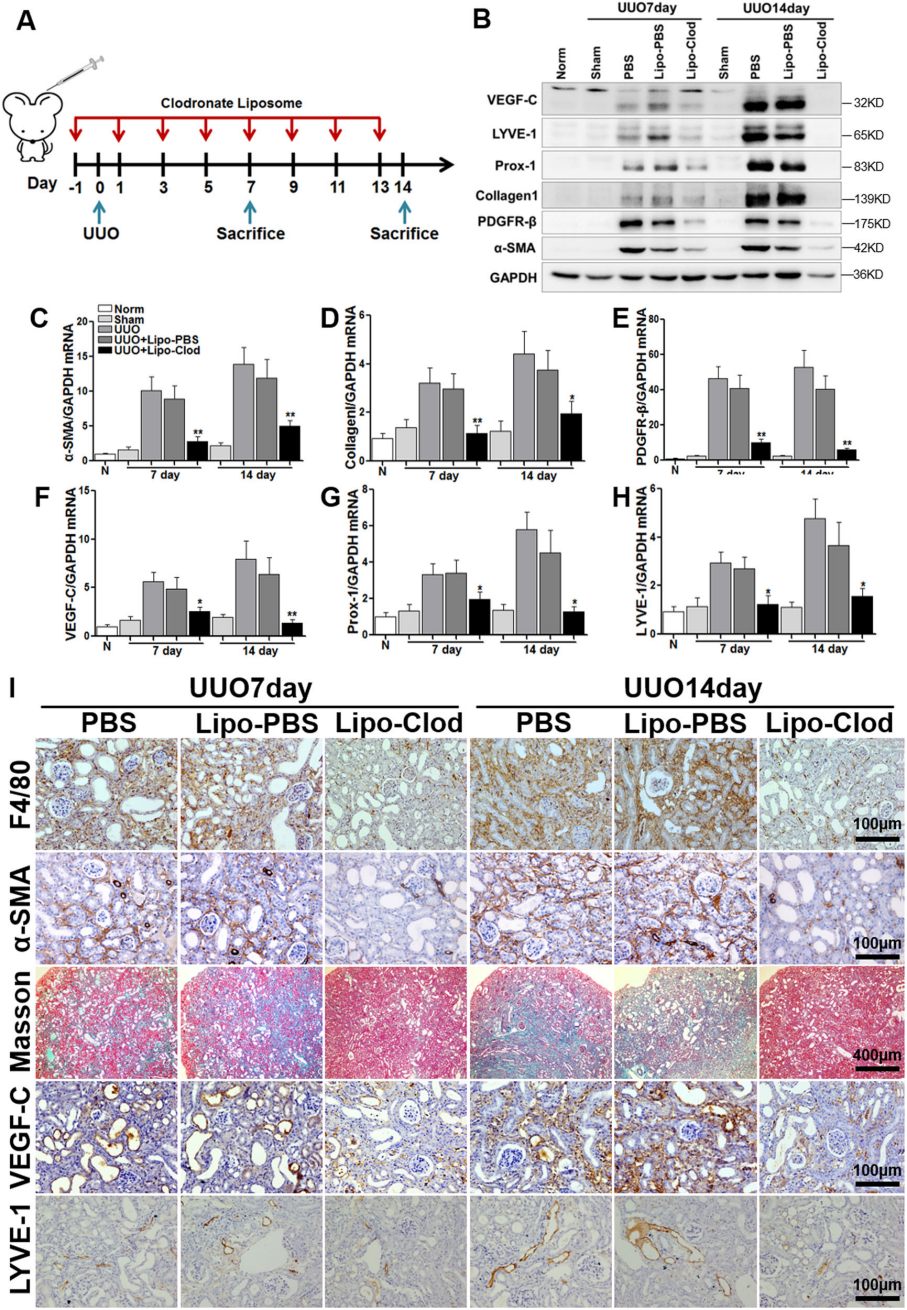
Macrophage depletion by clodronate liposomes downregulated lymphangiogenesis in UUO mice. **A** Schematic showing macrophage depletion by clodronate liposomes: 200 µl of clodronate liposomes (Lipo-Clod) or control liposomes (liposomes containing PBS, Lipo-PBS) were injected by intraperitoneal injection 1 day before the operation (UUO), and then 100 µl of clodronate liposomes or PBS liposomes were injected every other day until the mice were sacrificed. **B** Representative western blot showing renal fibrosis (Collagen1, PDGFR-β, and α-SMA) and lymphangiogenesis (Prox-1, LYVE-1, and VEGF-C) in the different groups. Relative mRNA expression of α-SMA (**C**), collagen 1 (**D**), PDGFR-β (**E**), VEGF-C (**F**), Prox-1 (**G**), and LYVE-1 (**H**) in all groups were measured by real-time PCR; *p < 0.05, **p < 0.01 versus the UUO + Lipo-PBS group. **I** Representative images showing Masson staining (100×) and immunohistochemical staining (400×) of renal fibrosis (α-SMA), macrophage infiltration (F4/80), and lymphangiogenesis (LYVE-1 and VEGF-C). Norm indicates normal control mice without any treatment, Sham indicates sham-operated mice vs UUO mice, UUO indicates unilateral ureteral obstruction, PBS indicates intraperitoneal injection of PBS, Lipo-PBS indicates intraperitoneal injection of PBS liposomes as a control, and Lipo-Clod indicates intraperitoneal injection of clodronate liposomes. *N* = 6/group.

To clarify the specific role of macrophage infiltration in lymphangiogenesis, we depleted macrophages from the UUO model using clodronate liposomes (Fig. 2A). Clodronate liposomes can be phagocytosed by macrophages. Once inside lysosomes, clodronate is released, and when the concentration reaches a certain level, macrophage apoptosis is induced, and macrophages are depleted^12^. In this experiment, intraperitoneal injection of clodronate liposomes was performed the day before the UUO operation and once every other day after the operation (Lipo-Clod). Liposomes containing PBS (Lipo-PBS) and pure PBS (PBS) were used as controls. As shown in Figs. 2I and S3, after the application of clodronate liposomes in UUO mice (Lipo-Clod group), macrophages and T cells infiltration were significantly reduced, and there was no significant difference in the number of innate macrophages in normal kidneys. Masson staining showed reduced fibrosis. IHC showed that the expression of α-SMA, VEGF-C, and LYVE-1 was significantly downregulated by clodronate liposomes (Fig. 2I). Western blotting (Fig. 2B) and real-time PCR (Fig. 2C–H) results also illustrated that renal fibrosis (α-SMA, PDGFR-β, collagen1) and lymphangiogenesis (VEGF-C, LYVE-1) in the Lipo-Clod group were significantly lower than those in the Lipo-PBS group. Unfortunately, a large number of ADR mice died after liposome-mediated depletion of macrophages, and so the relevant results were incomplete. These results suggest that early clearance of macrophages could downregulate renal fibrosis and reduce the expression of lymphangiogenesis and growth factors, indicating that macrophages played a vital role in promoting lymphangiogenesis.

### Lymphatic vessel markers are coexpressed with macrophages in vivo and in vitro

**Fig. 3:**
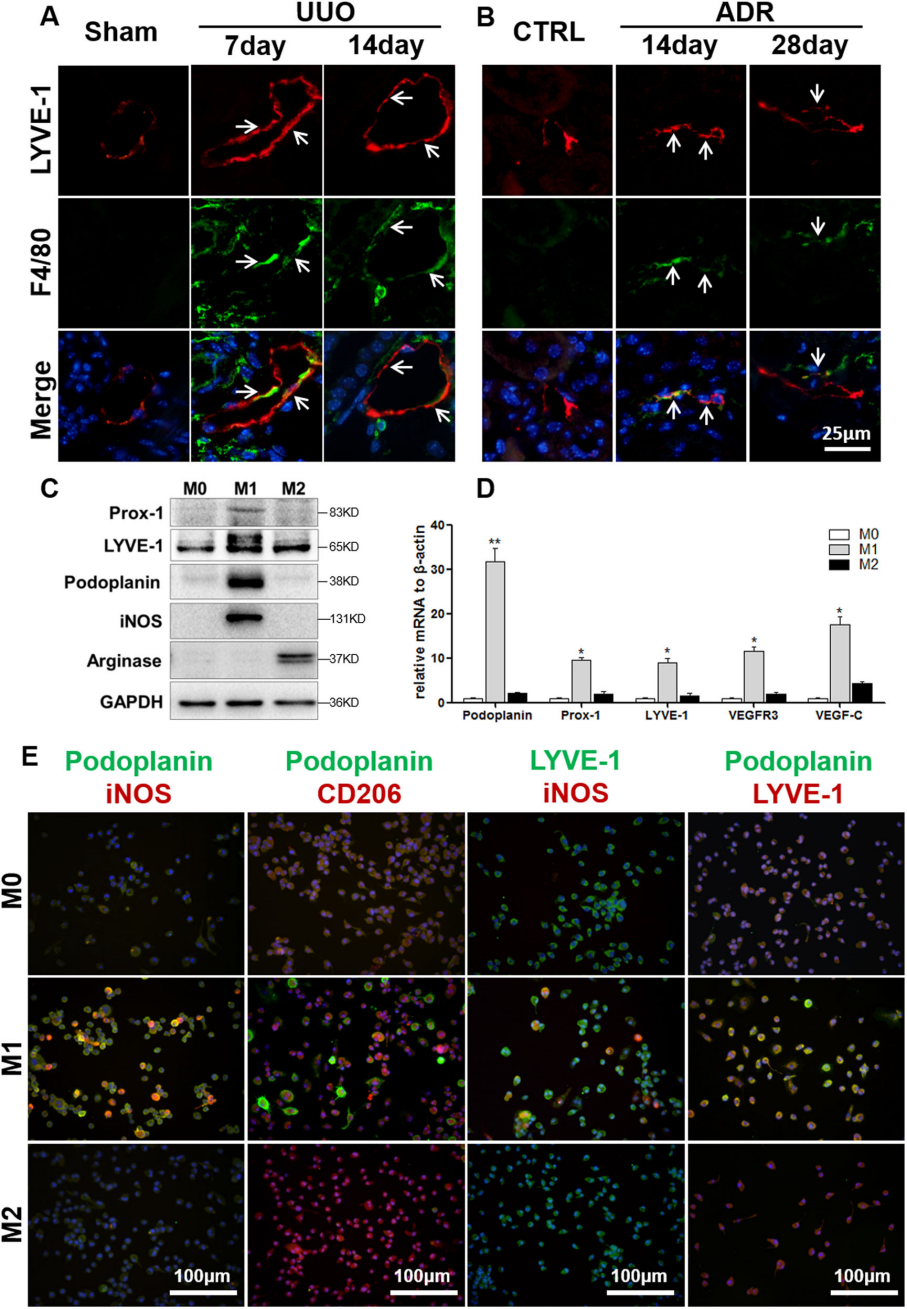
Coexpression of macrophages and lymphatic vessel cells in vivo and in vitro. Colocalization of F4/80^+^ macrophages and LYVE-1^+^ lymphatic vessels in UUO mice (400×) (**A**) and ADR mice (400×) (**B**). **C** Primary bone-marrow-derived macrophages (BMDMs) were isolated and induced to form M0, M1, and M2 macrophages in vitro, and western blot analysis suggested that compared to M0 and M2 cells, M1 macrophages predominantly expressed lymphatic vessel markers (Prox-1, LYVE-1, and podoplanin). **D** Relative mRNA expression of podoplanin, Prox-1, LYVE-1, VEGFR3, and VEGF-C was measured in BMDM subpopulations by real-time PCR; *p < 0.05, **p < 0.01 versus M0 or M2 macrophages. **E** Immunofluorescence results showed that M1 markers (inos) colocalized more with lymphatic vessel markers (LYVE-1 and podoplanin) than M0 and M2 (CD206), and lymphatic vessel markers (LYVE-1 and podoplanin) tended to be expressed in M1 macrophages (400×). Merged representative images also show nuclei (DAPI in blue). *N* = 6/group.

To determine whether macrophages can be directly differentiated into LECs in renal fibrosis, immunofluorescence double-labeling of macrophages (F4/80) and lymphangiogenesis markers (LYVE-1) was conducted to analyze colocalization in UUO (Fig. 3A) and ADR (Fig. 3B) mice, and the results showed that some LYVE-1^+^ lymphatic vessels were also positive for F4/80. This finding indicates that there may be some degree of homology between lymphatic vessels and macrophages.

To further clarify the potential mechanism of macrophage transdifferentiation into LECs, primary bone-marrow-derived macrophages (BMDMs) were harvested, and resting (M0) macrophages were activated to form classically activated (M1) macrophages and alternatively activated (M2) macrophages with LPS/IFN-γ and IL-4/IL-13, respectively. The identification of BMDMs is shown in Fig. S2. iNOS, TNF-α, CD86, and MHC-II are specific markers for M1 macrophages, and arginase, CD206, YM-1, FIZZ-1, and dectin-1 are specific markers for M2 macrophages. Western blotting (Fig. 3C) and real-time PCR (Fig. 3D) results showed that M1 macrophages expressed significantly higher levels of the lymphangiogenic markers Prox-1, LYVE-1, and Podoplanin and the lymphangiogenic growth factor VEGF-C than M0 or M2 macrophages. Immunofluorescence analysis of primary macrophages (Fig. 3E), showed that Podoplanin was expressed in M1 macrophages and colocalized with iNOS but not CD206. LYVE-1 was also preferentially expressed in M1 macrophages. Moreover, Podoplanin and LYVE-1 were both dominantly expressed and colocalized on M1 macrophages. These results indicate that some macrophages express lymphatic endothelial cell markers, suggesting that macrophages transdifferentiate into LECs. Among the three types of macrophages, the M1 subtype, which is classically activated, expresses more lymphatic endothelial cell markers and is more likely to be transformed into LECs.

### VEGF-C/VEGFR3 signaling promotes M1 differentiation

**Fig. 4:**
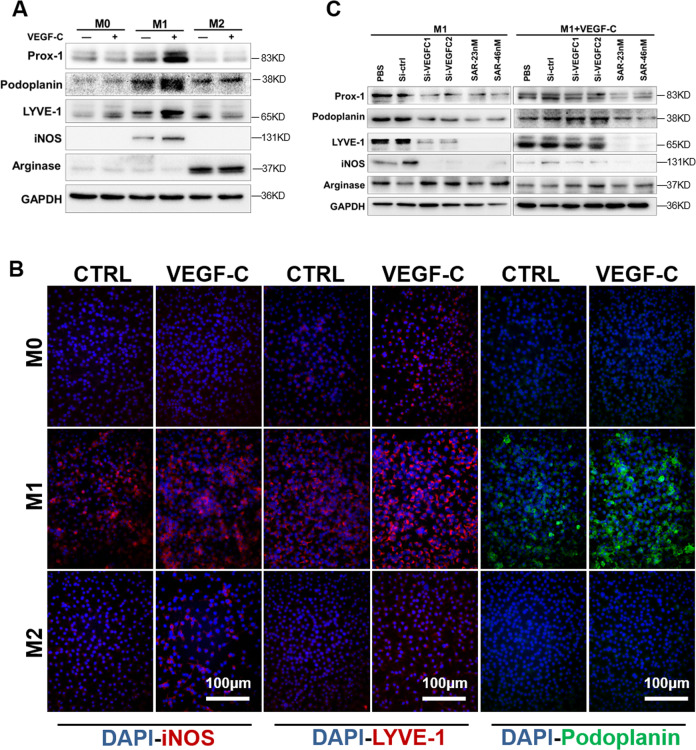
VEGF-C stimulated M1 further polarization and transdifferentiation into lymphatic vessel cells. **A** Primary BMDMs subtypes were stimulated by VEGF-C in vitro, and then the expression of macrophage activation markers (iNOS for M1, arginase for M2) and lymphatic vessel markers (Prox-1, LYVE-1, and podoplanin) were measured by western blotting. **B** In vitro, primary BMDMs were analyzed by immunofluorescence for iNOS, LYVE-1, and podoplanin expression after VEGF-C stimulation or control treatment (CTRL) (400×). **C** M1 macrophages were treated with VEGF-C, siRNA for VEGF-C (Si-VEGF-C), and the VEGFR3 inhibitor SAR131675 (SAR-23/46 nM), and then the protein expression of iNOS, arginase, and lymphatic vessel markers (LYVE-1 and Prox-1) was measured.

As a classic lymphatic growth factor, VEGF-C is abundantly expressed in the fibrosis model, and both tubules and interstitial cells secrete VEGF-C (Figs. 1A and S1A). Thus, interstitial macrophage differentiation in the microenvironment must be affected. Western blotting analysis showed that VEGF-C stimulated an increase in iNOS expression in M1 macrophages and increased the expression of the Prox-1, Podoplanin, and LYVE-1 (Fig. 4A). Immunofluorescence results also confirmed that VEGF-C stimulated an increase in iNOS expression on M1 macrophages and upregulated the expression of LYVE-1 and Podoplanin on M1 macrophages (Fig. 4B); VEGF-C acts primarily by binding to receptors, mainly VEGFR3. Furthermore, in response to VEGF-C-siRNA (si-VEGFC1 and si-VEGFC2) and different concentrations of the VEGFR3 inhibitor SAR131675 (SAR-23 nM and SAR-46 nM), iNOS expression decreased on M1 macrophages, and Prox-1, Podoplanin, and LYVE-1 expression also decreased; after VEGF-C was added, the effect of siVEGF-C was reversed, but the effect of SAR131675 persisted (Fig. 4C). These results suggest that VEGF-C further promotes an increase in the M1 phenotype while increasing lymphatic vessel marker expression, which can be eliminated by VEGFR3 inhibitors or siVEGF-C.

### Primary BMDMs formed tube-like structures in vitro

**Fig. 5:**
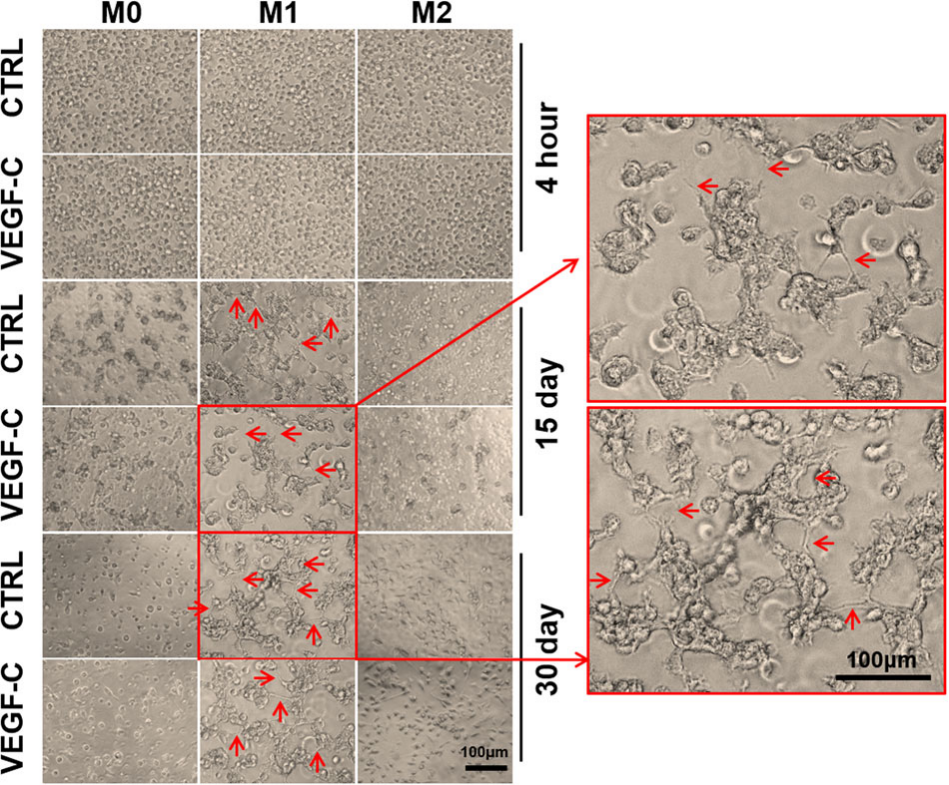
Primary BMDMs formed tube-like structures in vitro. Behavior of BMDMs with or without VEGF-C treatment in a tube formation assay containing 2 × 10^5^ cells/well in Matrigel for 4 h, 15 days, and 30 days (200×).

Although macrophages can express lymphatic vessel markers, whether these cells can be truly transformed into lymphatic vessels needs further verification. Three subtypes of macrophages (with or without VEGF-C stimulation) were cultured in a growth factor reduced Matrigel-3D culture system and observed daily, with medium exchange every other day (Fig. 5). M0 and M2 cells showed no special effects during 3D culture, and the cells dispersed away from each other after the start of culture; the ability of M0 macrophages to cross the matrix was weak, and during the 30 days of culture, the number of cells decreased, the state of the cells became worse, and some cells became apoptotic. M2 macrophages had a strong ability to cross the matrix and remained in a good state until the 30th day. These cells gradually crossed the matrix to the bottom of the plate, showed a partially adherent macrophage morphology and extended pseudopodia. M1 cells were close to each other throughout the culture period, and they were regularly arranged. After 15 days, some cells formed clusters and protruded from the bud-like structure of endothelial cells. As the culture time increased, the numbers of cell clusters and budding cells increased, and some sprouts formed a connection, gathering into tube-like structures. After 30 days of culture, M1 cells were still in good condition, and the tubular structures and cell clusters were more obvious than earlier in the culture period. Tube-like structures could also be produced by treatment of M1 cells with VEGF-C. However, due to the differences in the tube-like structures formed by macrophages compared to ordinary endothelial cells, quantitative analysis of the tubes could not be performed by conventional methods. Cell density was very important in this experiment. Too few or too many cells prevented the formation of tubular structures.

### Primary BMDMs formed tube-like structures in vivo

**Fig. 6:**
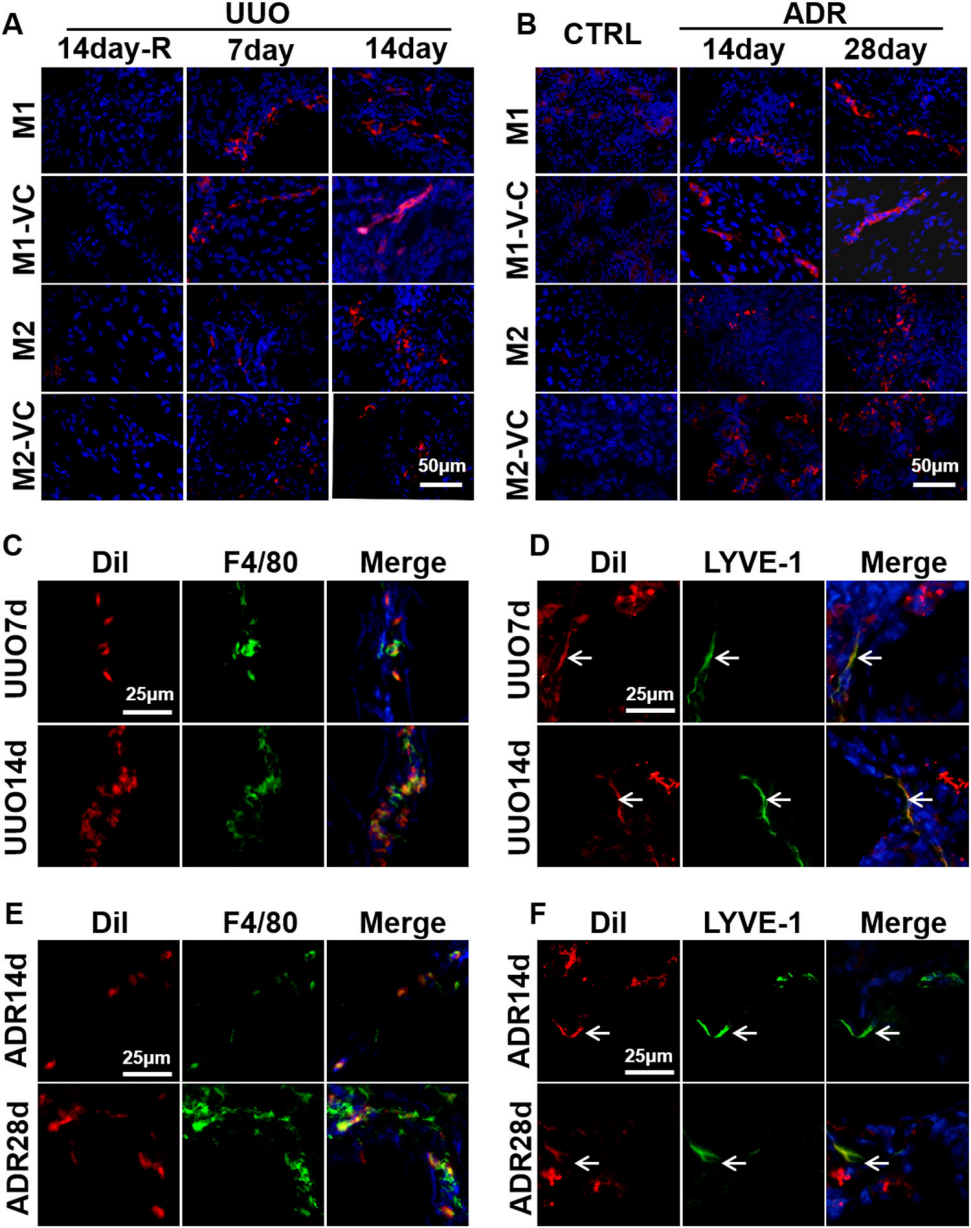
Primary BMDMs formed tube-like structures in vivo. DiI (red)-labeled BMDMs (M1, M2) were injected into UUO mice (**A**) and ADR mice (**B**) through the tail vein. After sacrifice, the mouse kidney sections were directly analyzed by fluorescence microscopy, and the operated kidney tissue was compared with the contralateral untreated kidney tissue. ADR mice were compared with saline-treated mice. Blue indicates DAPI-stained nuclei (400×). **C**–**F** Transferred DiI-M1 (red) cells were analyzed for coexpression of F4/80 (**C**, **E**) and LYVE-1 (**D**, **F**) in UUO (**C**, **D**) and ADR (**E**, **F**) mice by immunofluorescence (400×). *N* = 6/group.

To demonstrate the potential of macrophages to generate lymphatic vessels in vivo, adoptive transfusion of primary macrophages was performed. Primary M1 and M2 BMDMs (with or without VEGF-C stimulation) were labeled with the red fluorescent cell membrane probe DiI and adoptively transferred into UUO day 1 or ADR day 5 mice via the tail vein. No fluorescent cell infiltration was observed in the contralateral nonoperative kidney (UUO-14-day-R) in the UUO model and ADR control group (CTRL). However, in UUO and ADR kidneys, DiI red fluorescence was observed. In the M1-DiI group (with or without VEGF-C stimulation), in addition to scattered red fluorescent cells, a few red fluorescent cells were arranged in clusters, and some formed tube-like structures that were continuous or discontinuous. In the M2-DiI group (with or without VEGF-C stimulation), only scattered red fluorescent cells were observed (Fig. 6A, B). However, in this in vivo experiment, M1 cells treated with VEGF-C did not form more tube-like structures than M1 cells (Fig. 6A, B), which might be related to the presence of more VEGF-C in the microenvironment of renal fibrosis. Further fibrotic kidney tissue frozen sections from DiI-M1-transfused mice were stained for F4/80 (Fig. 6C, E) or LYVE-1 (Fig. 6D, F). It was found that the red fluorescence contained with F4/80 in UUO and ADR kidneys, indicating that these cells were macrophages. However, the colocalization of F4/80 with DiI was not consistent, suggesting that macrophage markers also changed during transformation and differentiation. In the two fibrosis models, the red fluorescence partially colocalized with LYVE-1 and occurred mostly in the tube-like structures or the neatly arranged cell clusters. This finding indicated that M1 cells might join the lymphatic structure in vivo and acquire a lymphatic endothelial cell phenotype.

### Autophagy participates in VEGF-C/VEGFR3 signaling-related macrophage differentiation

**Fig. 7:**
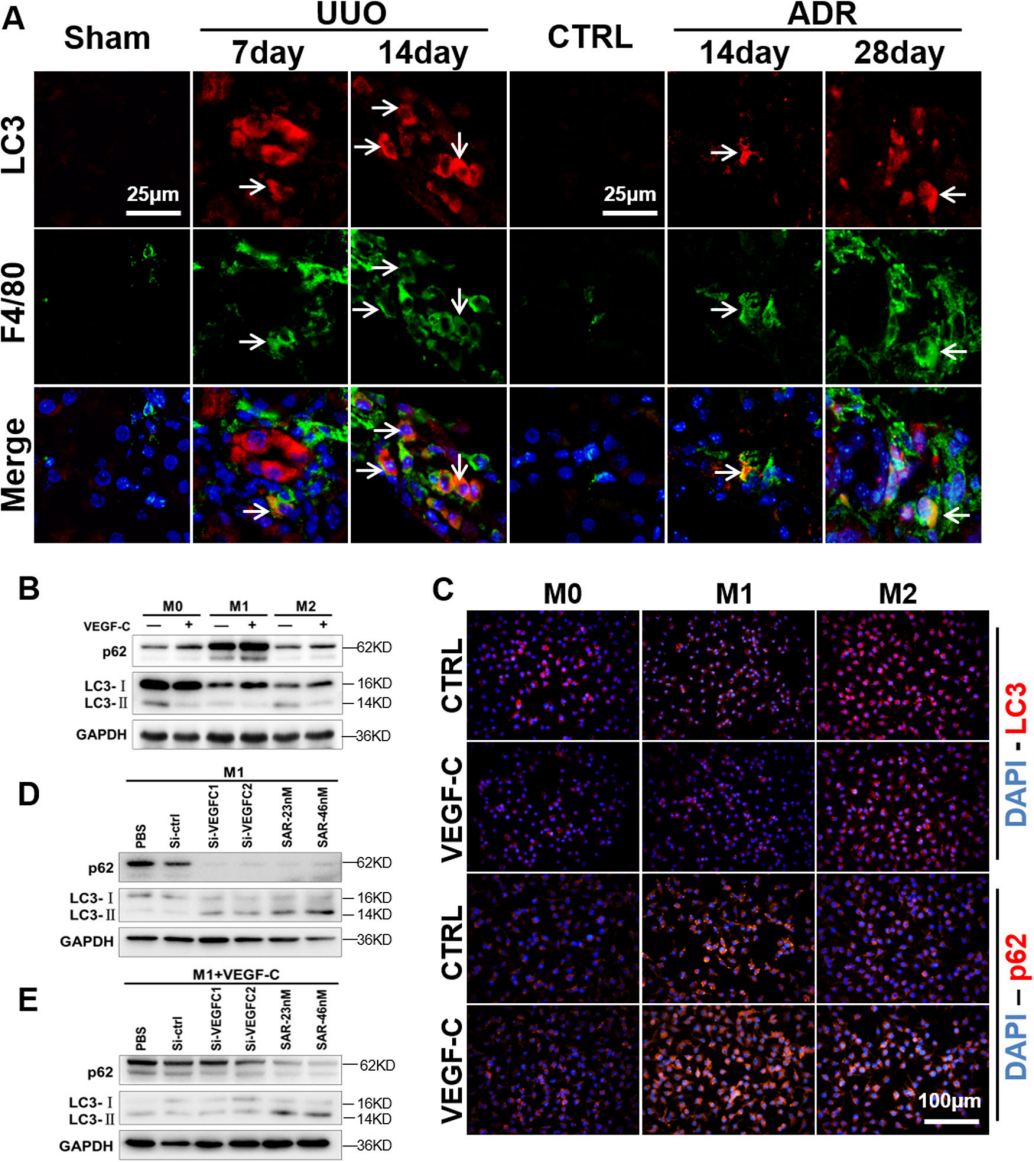
Autophagy is involved in M1 transdifferentiation into LECs. **A** Infiltrated macrophages (F4/80) in UUO and ADR mice exhibited different autophagy levels (LC3) (400×). **B** Primary BMDMs (M0, M1, and M2) were treated with or without VEGF-C, and the protein expression of autophagy-related markers (LC3 and p62) was measured by western blotting. **C** Immunofluorescence was used to show the effect of VEGF-C on autophagy in BMDM subtypes (400×). **D**, **E** M1 cells were treated with VEGF-C, siRNA targeting VEGF-C (Si-VEGF-C) and/or the VEGFR3 inhibitor SAR131675 (SAR-23/46 nM), and the protein expression of autophagy markers (LC3 and p62) was measured by western blotting. *N* = 6/group.

Furthermore, we examined macrophage autophagy to explore the mechanism of macrophage phenotypic transition. F4/80 and LC3 immunofluorescence staining were performed on UUO and ADR mice (Fig. 7A). We observed that autophagy was widespread in the fibrosis model, and there were different degrees of autophagy in the glomeruli, tubules, and mesenchymal cells. The same effects were observed in macrophages. In vitro, the autophagy level was also measured in primary BMDMs, and the autophagy level in M1 macrophages was lower than that in M2 and M0 macrophages, with reduced LC3-II/I expression and increased p62 expression (Fig. 7B, C). VEGF-C downregulated LC3-II/I expression and upregulated p62 expression in BMDMs. After the application of VEGF-C-siRNA and the VEGFR3 inhibitor SAR131675 (Fig. 7D, E), M1 macrophage LC3-II/I expression was increased and p62 expression was decreased. In the presence of exogenously added VEGF-C, VEGF-C-siRNA had no effect, but the effect of SAR131675 persisted. These results suggest that VEGF-C/VEGFR3 signaling may change the macrophage autophagy. Earlier in Fig. 4, we found that VEGF-C/VEGFR3 signaling activation promoted M1 polarization and transdifferentiation into LECs. And studies have shown that macrophages activation was related to autophagy^11^. Combined with Fig. 4, Fig. 7, and related literatures, we have reason to believe that VEGF-C/VEGFR3 signaling may change the macrophage phenotype via autophagy.

### Rapamycin decreases M1 polarization via autophagy induction

**Fig. 8:**
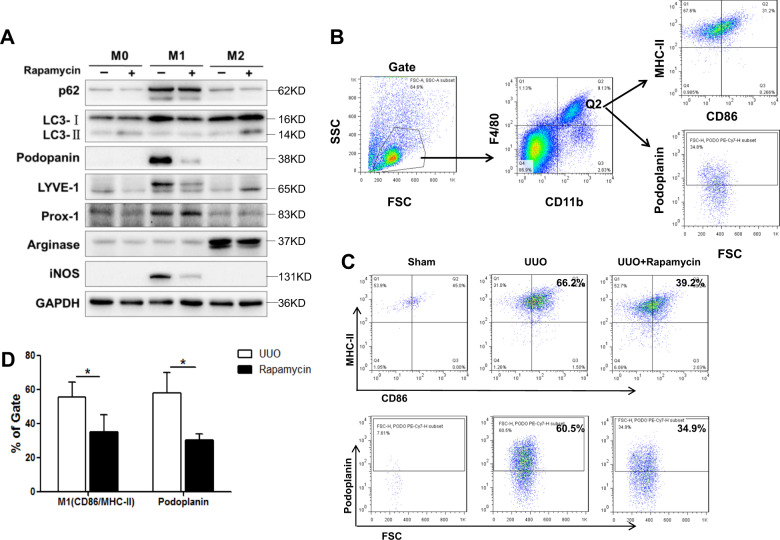
Rapamycin upregulated macrophage autophagy and ultimately downregulated macrophage transdifferentiation into LECs. **A** Autophagy was induced in primary BMDMs by rapamycin, and the expression of macrophage polarization markers (arginase and iNOS) and lymphatic vessel markers (podoplanin, LYVE-1, and Prox-1) was measured by western blotting. **B**–**D** Rapamycin affected macrophage polarization and transdifferentiation in UUO mice. Macrophage activation and the expression of lymphatic vessel markers were measured by flow cytometry. Panel **B** shows the gating method: macrophages were double-positive for F4/80 and CD11b, of which CD86^+^MHC-II^+^ cells were M1 and podoplanin^+^ cells were considered LECs. The upper panel in **C** shows that rapamycin decreased M1 polarization in UUO mice, and the lower panel in **C** shows that rapamycin also decreased lymphatic vessel marker expression in infiltrated macrophages in UUO mice. D is the statistical graph of the data in (**C**). *p < 0.05. *N* = 6/group.

To confirm whether modulating the state of autophagy in macrophages affected macrophage phenotype, rapamycin was used as an autophagy inducer. In vitro, after rapamycin treatment, LC3-II/I expression in M1 macrophages was elevated, p62 protein was degraded, iNOS expression was significantly weakened, and expression of the Prox-1, Podoplanin, and LYVE-1 were all decreased (Fig. 8A). In vivo, UUO mice were also treated with rapamycin, and the kidney cells were harvested for flow cytometric analysis (Fig. 8B, C). After regular gating, macrophages were detected by the expression of F4/80 and CD11b, and then the ratio of M1 cells and LECs was detected in the macrophage population. In UUO day 10 samples, the proportion of M1 macrophages was 55.7 ± 8.9% and that of Podoplanin^+^ cells were ~58.2 ± 11.7%. After administration of rapamycin to UUO mice, the proportion of M1 macrophages decreased to 35.2 ± 9.9%, while that of Podoplanin^+^ cells in the total macrophage population was ~30.4 ± 3.7%, which was significantly lower than that of the UUO group (*p* < 0.05) (Fig. 8D). This finding indicated that the autophagy inducer rapamycin inhibited the M1 differentiation of macrophages and weakened the ability of macrophages to differentiate into LECs in UUO, suggesting the key role of autophagy in macrophage differentiation.

**Fig. 9:**
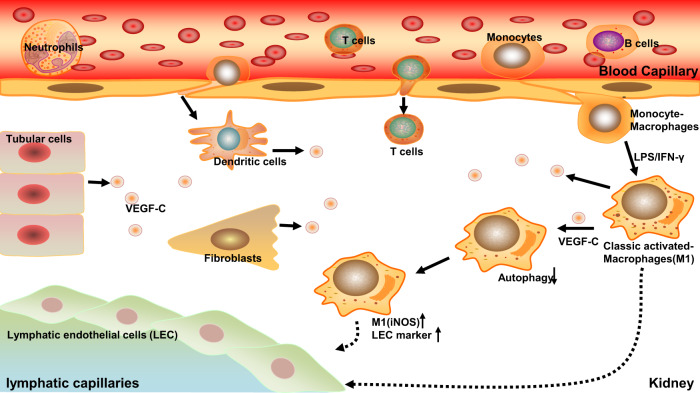
Schematic diagram of how macrophages participate in lymphangiogenesis in renal fibrosis. When renal fibrosis is initiated, circulating inflammatory cells are chemically induced to home to the tissue, including macrophages, T cells, dendritic cells, and so on. Macrophages are polarized to M0, M1, and M2 phenotypes. Among them, M1 macrophages can preferentially transdifferentiate into LECs. During this process, the lymphatic growth factor VEGF-C, which is produced by infiltrated inflammatory cells, tubular cells, and other interstitial cells, suppresses M1 macrophage autophagy, thus upregulating M1 marker expression and polarization and ultimately promoting macrophage differentiation into LECs.

In conclusion, the inflammatory microenvironment of renal fibrosis recruited macrophages, and the lymphangiogenic growth factor VEGF-C/VEGFR3 downregulated macrophage autophagy, promoting differentiation into M1 macrophages and further transdifferentiation into LECs (Fig. 9).

## Discussion

As early as 2009, studies indicated that lymphangiogenesis was common in the progression of tubulointerstitial fibrosis^4^. Subsequently, Hasegawa et al. showed that induction of lymphangiogenesis using VEGF-C ameliorates renal fibrosis, indicating a negative regulatory effect of lymphangiogenesis on fibrosis^13^. Others found that inhibition of lymphangiogenesis by VEGFR3 inhibitors or PPARα agonists ameliorates diabetic nephropathy^14,15^. Work by Pei also elucidated the role of lymphangiogenesis in promoting renal fibrosis^16^. Although the purpose of lymphangiogenesis is not completely determined and understood, there is no doubt about the importance of lymphangiogenesis in renal fibrosis. Our research also confirmed that lymphangiogenesis was positively correlated with renal fibrosis (Figs. 1 and S1). However, the sources of lymphatic progenitor cells and the way that lymphatic vessels are formed have generated much attention and debate.

The canonical lymphatic vessel generation is de novo vascular formation and sprouting during the embryonic period and branching out from existing vessels during adulthood^17^. The updated concept focused on lymphatic endothelial cell progenitors (LECPs), which mobilize to injured tissues, inflamed tissues, or tumors, where they undergo transdifferentiation and integration into the existing lymphatic network^10,17^. Hematopoietic stem cells (HSCs)^18^, mesenchymal stem cells (MSCs)^19^, adipose-derived stem cells (ADSCs)^20^, and myeloid stem or progenitor cells^21^ have been reported to be LECPs. Current lineage-tracing studies revealed LECPs diversity in different organs. The origin of lymphatic cells in renal disease has not been well studied thus far and is not completely understood. Our previous study found that lymphangiogenesis after renal injury was mainly caused by local hyperplasia of pre-existing LECs^16^. However, a study on human renal transplantation revealed the contribution of circulating cells, especially bone-marrow-derived myeloid cells, to lymphangiogenesis^8^.

In this study, there was a positive correlation between renal fibrosis-associated lymphangiogenesis and macrophage infiltration, and macrophage depletion by clodronate liposomes downregulated lymphangiogenesis in UUO mice, suggesting the important role of macrophages in lymphangiogenesis. Macrophages may participate in lymphangiogenesis in two ways: indirect involvement by secreting lymphangiogenic growth factors and by direct transdifferentiation into lymphatic vessels^22^. Next, we verified the colocalization of macrophages and lymphatic markers in fibrotic kidneys and identified the latter pathway in renal fibrosis. However, this conclusion is inconsistent with findings in some other organs^23^, and only a few lymphatic vessels were colocalized with macrophages in our study. LYVE-1 is also expressed on the surface of some macrophage subtypes in tumors^24^; therefore, more in vivo or in vitro evidence about whether macrophages are LECPs is needed. Macrophages are heterogeneous and can be induced to differentiate into multiple subtypes in different tissue microenvironments. Typically, macrophages can be polarized into M0 (resting macrophages), M1 (classically activated), and M2 (alternatively activated) phenotypes^25^. Most studies identified CD11b^+^ myeloid cells as LECPs or cytokines from activated macrophages as lymphangiogenic factors. However, the question of which subtype of macrophages can promote lymphangiogenesis has been controversial. In previous studies, M2 tumor-associated macrophages (TAM) were found to be significantly related to the density of lymphatic vessels adjacent to tumors, while M1-TAM had nothing to do with these parameters^26,27^. IL-10 could also induce corneal lymphatic vessels through macrophages^28^. Some factors secreted by M2 also promoted lymphangiogenesis^29^. But it seems contradictory to this; studies have found that CLEVER-1/Stablin-1 positive M2 had nothing to do with Podoplanin-positive lymphatic vessel density^30^, while Th2-related cytokines (M2-related) could inhibit lymphangiogenesis^31^. TNF-α, the product of M1, also induced lymphangiogenesis^32^. A study by Kim, Y also showed that expression of the M1 marker iNOS paralleled expression of the lymphatic-related factors VEGF-C and VEGFR3 in RAW264.7 cells^15^. The inconsistency of these research results may be related to different tissues, cell lines, and stimulation conditions. In addition, there were few studies discussing the direct relationship between M1/M2 and lymphangiogenesis. For the first time, we directly compared these subtypes and found that M1 macrophages predominantly expressed lymphatic markers, providing direct evidence for the above issues. To verify that M1 macrophages have the characteristics and functions of endothelial cells, we performed tube formation experiments in vitro and adoptive transfer experiments in vivo. M1 macrophages accumulated and sprouted in Matrigel, connected, and finally formed tube-like structures, while M2 macrophages were scattered against the wall of the plate. In vivo, many M1 and M2 macrophages were both recruited to the fibrotic kidney after adoptive transfusion. Small numbers of M1 macrophages were distributed in the form of cell clusters, and some cells formed thin-walled lumens, while M2 macrophages were only dispersed. Therefore, M1 macrophages can transdifferentiate into LECs in vivo and in vitro with a low conversion rate. It is well known that M1 macrophages promote the inflammatory response to clear pathogens, and M2 macrophages eliminate inflammation to promote repair^25,33^. Therefore, the finding that M1 macrophages promote lymphangiogenesis is consistent with our previous findings that neovascular lymphatics promote the renal inflammatory response^16^.

Although there is evidence that VEGF-A/C/D and VEGFR2/3 are all involved in lymphangiogenesis, VEGF-C/VEGFR3 remains the most classic pathway^34,35^. Inhibition of this pathway leads to changes in lymphatic vessel structure and function, and so its role in macrophage transdifferentiation into LECs needs to be explored. We found that VEGF-C further increased the expression of iNOS in M1 macrophages, as well as the expression of lymphatic vascular markers. In the tube formation assay, the effect of VEGF-C on M1 macrophages was not obvious because of statistical difficulties caused by irregular lumen formation. Adoptively transferred VEGF-C-treated M1 macrophages did not form more tubes than normal M1 macrophages, and high levels of VEGF-C in the renal fibrosis microenvironment may account for this effect. The expression of Arginase in M2 macrophages was also slightly increased after VEGF-C treatment, but this will not be discussed further because of the low expression of lymphatic vascular markers in M2 macrophages. In 2014, D’Alessio reported that the VEGF-C/VEGFR3 pathway induced inflamed colonic expression of hybrid M1-M2 markers^36^; recently, a study on intrarenal lipotoxicity showed that increased VEGF-C correlated with enhanced expression of the M1 phenotype in vivo and in RAW264.7 cells^15^. Our results are similar to these two studies. However, the mechanism underlying the effect of VEGF-C/VEGFR3 on macrophage phenotype remains unknown.

Autophagy is a highly conserved degradation and recycling process that has been reported to play an important role in macrophage polarization^37^. Different studies have obtained controversial results in the context of different pathological environments, macrophage cell lines, and stimulating factors^38–44^. In our study, primary M1 macrophages exhibited a lower level of autophagy than M2 macrophages. The macrophages infiltrating in the early stage of chronic kidney disease are mainly the M1 phenotype, and the number and proportion of M2 cell infiltration increase as the disease progresses. Therefore, we observed an increase in the number and proportion of macrophages with a high degree of autophagy at the late stage of the mice model, which was probably related to the increase in the proportion of M2 cells. We also found that VEGF-C could inhibit autophagy in macrophages and then further differentiate macrophages into the M1 phenotype and lymphatic vascular cells. This is contrary to the effect observed in tumor cells but similar to the results observed in diabetic mice^42,45^. To date, studies on VEGF-C-mediated regulation of autophagy have been very limited. In 2013, Stanton et al. discovered for the first time that VEGF-C/NRP-2 axis-activated autophagy helps tumor cells resist treatment^45^. Until recently, our team reported that the VEGF-C/NRP-2 axis can regulate renal tubular epithelial cell autophagy to repair damage^46^. While VEGFR3 is a coreceptor for NRP-2^47^, the present study reported for the first time that VEGF-C/VEGFR3 regulated macrophage autophagy, which in turn affected macrophage polarization and differentiation. To confirm the role of autophagy in VEGF-C/VEGFR3-mediated alteration of macrophage phenotype, we also used the mTOR inhibitor rapamycin to induce autophagy in macrophages and found that rapamycin reversed the effect of VEGF-C. The ratio of M1 macrophages in UUO kidney tissue was reduced, the proportion of macrophages with lymphatic vessel markers was reduced, and the expression of iNOS in primary M1 macrophages was downregulated. Rapamycin has been found to inhibit lymphangiogenesis in tumors^48^, reduce renal fibrosis^49^, and reduce rejection after transplantation^50^. Combined with our results, it can be hypothesized that rapamycin increases the autophagy level of macrophages, inhibits M1 macrophage differentiation, and downregulates lymphangiogenesis, thereby reducing inflammatory responses in acute and chronic kidney diseases.

In conclusion, this study explored the relationship between infiltrating macrophages in renal fibrosis and the source of lymphangiogenesis through in vivo and in vitro experiments. We found that M1 macrophages were more likely to transdifferentiate into LECs than M0 or M2 macrophages. Activation of the VEGF-C/VEGFR3 pathway can promote phenotypic changes in M1 macrophages and transdifferentiation of these cells into LECs through the downregulation of autophagy. These results provide a new perspective for understanding the role and significance of macrophages, lymphangiogenesis, and autophagy in renal fibrosis and provide a new theory for renal fibrosis intervention.

## Materials and methods

### Animal model

Six-week-old male C57BL/6 mice and BALB/C mice were obtained from the HuaFukang Experimental Animal Center (Beijing, China). The mice were provided a standard laboratory diet and water in the animal facility of Tongji Medical School. After a minimum of 7 days of acclimation, the mice were randomly allocated to different groups. Then they were used to establish the unilateral ureteral obstruction (UUO) and adriamycin nephropathy model (ADR) models. For the UUO model, C57BL/6 mice were anesthetized, and a lateral incision was made on the back. Then, the left ureter was carefully separated and ligated with 4.0 silk. The operation for mice in the Sham group was the same except that ureteral ligation was not performed. For the ADR model, adriamycin (Sigma-Aldrich, USA) was diluted to 1 mg/ml, and BALB/C mice were injected with adriamycin (14 mg/kg) via the tail vein. Mice in the control group (CTRL) were injected with equal amounts of saline. The mice were sacrificed by cervical vertebra dislocation, and renal tissues were collected. The harvested kidneys were fixed in 4% formalin for 24 h, dehydrated in a graded series of alcohol, and then embedded in paraffin. The remaining tissues were frozen in liquid nitrogen for subsequent testing. In some experiments, UUO mice were given intraperitoneal injection of rapamycin (Selleck, USA; 2 mg/kg/day) the day before the operation and were killed after 10 days. All experimental procedures were performed in accordance with internationally accepted laboratory principles.

### Preparation of primary BMDMs

A standard BMDMs preparation method was used. Briefly, under aseptic conditions, the mice were sacrificed by cervical dislocation and disinfected. The femur and tibia were quickly removed and rinsed to isolate primary cells. Then, the cells were filtered and cultured for 7 days in DMEM (HyClone, USA) with 10% heat-inactivated fetal bovine serum and 20% L929 cell-conditioned medium. In some experiments, VEGF-C (Peprotech, USA), siRNA-VEGF-C, the VEGFR3 inhibitor SAR131675 (SAR-23 nM and SAR-46 nM) (Selleck, USA), or rapamycin (Selleck, USA) was added at the same time.

### VEGF-C silencing with siRNA

The specific siRNA and negative control siRNA oligonucleotides were purchased from RiboBio (China). BMDMs were transfected with siRNA by using Lipofectamine RNAi MAX (Invitrogen, USA) according to the standard protocol on the 6th day of culture. The medium was changed, and other factors were added 24 h after transfection. The silencing efficiency was tested by real-time PCR.

### Adoptive transfer of BMDMs

BMDMs were prepared as described above and M1/M2 macrophages, with or without treatment with VEGF-C stimulation, were harvested, washed, suspended and labeled with DiI (Beyotime, China). Then, 1 × 10^6^ cells were transferred via the tail vein to UUO mice on the day after the operation or to ADR/CTRL mice on the 5th day. UUO mice were sacrificed on the 7th and 14th days, and the operated and lateral kidneys were collected. ADR and CTRL mice were killed on the 14th and 28th days. The kidneys were embedded in OCT compound (Sakura, Japan) and fixed with acetone. For tube-like structure observation, sections were washed with ddH_2_O, blow-dried and sealed with ProLong Antifade reagent containing DAPI (Life Technology, USA). For identification of transferred cells, the sections were blocked with goat serum at room temperature, incubated with antibodies against F4/80 (Santa Cruz, USA) or LYVE-1 (Angiobio, USA) at 4 °C overnight, incubated with fluorescence-labeled secondary antibodies (Jackson ImmunoResearch) and sealed with ProLong Antifade reagent containing DAPI (Life Technology, USA).

### Immunohistochemistry

Three-micrometer paraffin-embedded renal sections were routinely dewaxed and hydrated, antigens were recovered by 10 mM citrate buffer (pH 6.0) at 98 °C for 10 min, endogenous peroxidase was blocked with 3% H_2_O_2_ for 15 min and nonspecific antigens were blocked with 10% goat serum for 30 min at room temperature. Sections were then incubated overnight with antibodies against α-SMA (Abcam, USA), F4/80 (Santa Cruz, USA), VEGF-C (Santa Cruz, USA), LYVE-1 (Angiobio, USA), followed by incubation with a horseradish peroxidase-conjugated secondary antibody and subsequently visualized with diaminoenzidine substrate and hematoxylin counterstaining. lymphatic vessels were measured according to Weidner’s classical method. Semi-quantification of other moleculars was conducted by imagePro plus. Randomly select 10 high-powered fields (400×), the proportion of the area of the positive area in the field was calculated by imagePro plus, the denominator was the area of the field. The average of the 10 fields was the semi-quantitative result of this slice.

### Immunofluorescence

Paraffin sections treated as above or BMDMs grown on coverslips in 12-well plate were incubated with primary antibodies against F4/80 (Santa Cruz, USA), LYVE-1 (Angiobio, USA), and LC3 (Sigma-Aldrich, USA), p62 (Abcam, USA), iNOS (Santa Cruz, USA), Podoplanin (Angiobio, USA), CD206 (Biolegend, USA) at 4 °C overnight, followed by incubation with fluorescence-labeled secondary antibodies (Jackson ImmunoResearch) for 45 min at 37 °C. In the end, slides were sealed by ProLong Antifade Reagents with DAPI and analyzed by Fluorescence microscope.

### Western blotting

Renal tissue and cells were harvested and lysed by RIPA lysis buffer (Wuhan Goodbio Technology, China) containing cocktail protease inhibitors (Wuhan Goodbio Technology, China) for 30 min on ice. The total protein was obtained by high-speed centrifugation at low temperatures. Protein concentrations were quantified by a BCA protein assay kit (Beyotime Institute of Biotechnology, China), and 30 µg protein was loaded, separated on 10% or 12% SDS-PAGE, and transferred to nitrocellulose membranes. Then membranes were blotted at 4 °C overnight with mouse anti-VEGF-C (Santa Cruz, USA; 1:250), rabbit anti-Prox-1 (Angiobio, USA; 1:1000), rabbit anti-LYVE-1 (Novus, USA; 1:1000), Hamster anti-Podoplanin (Angiobio, USA; 1:200), rabbit anti-Collagen1 (Novus, USA; 1:2000), rabbit anti-α-SMA (Abcam, USA; 1:4000), rabbit anti-PDGFR-β (Abcam, USA; 1:2000), mouse anti-iNOS (Santa Cruz, USA; 1:200), rabbit anti-Arginase (Santa Cruz, USA; 1:400), rabbit anti-LC3B (Sigma-Aldrich, USA; 1:1000), rabbit anti-p62 (Abcam, USA; 1:5000), mouse anti-GAPDH (Wuhan Goodbio Technology, China; 1:2000), then were incubated with HRP-conjugated anti-IgG (Jackson ImmunoResearch, USA; 1:4000), finally were detected by ECL (Pierce, USA). Image capture and analysis were conducted by Bio-RAD (USA).

### Quantitative real-time PCR

Total RNA was extracted from renal tissues and cells using TRIzol Reagent according to manufacturer’s protocol (Invitrogen, USA). One microgram of RNA was reverse-transcribed using the First Strand cDNA Synthesis Kit (Fermentas, USA). qPCR was performed using SYBR Master Mix (Fermentas, USA) and analyzed by Image Lab software (Bio-RAD, USA). mRNA expression levels were calculated using the 2^−ΔΔ^Ct approach and normalized to β-actin or GAPDH levels. The following primer sequences were used: mouse β-actin, forward 5′-CGTTGACATCCGTAAAGACC-3′, reverse 5′-AACAGTCCGCCTAGAAGCAC-3′; mouse GAPDH, forward 5′-AGGTCGGTGTGAACGGATTTG-3′, reverse 5′-GGGGTCGTTGATGGCAACA-3′; mouse Collagen1, forward 5′-GTCCTAGTCGATGGCTGCTC-3′, reverse 5′-CAATGTCCAGAGGTGCAATG-3′; mouse α-SMA, forward 5′-GGAGAAGCCCAGCCAGTCGC-3′, reverse 5′-AGCCGGCCTTACAGAGCCCA-3′; mouse PDGFR-β, forward 5′-GGGTCCGTTCCAGAAAATGT-3′, reverse 5′-GACAAGGGACCGGGGTCCAA-3′; Mouse Prox-1, forward 5′-CTACGAAACCCTTGCCCACT-3′, reverse 5′-AGCCACCCTCCATACCCATA-3′; mouse VEGF-C, forward 5′-GAGGAGCAGTTGCGGTCTG-3′, reverse 5′-TCCTGGTATTGAGGGTGGG-3′; mouse LYVE-1, forward 5′-AAAACTACGGTGCGATGCTTAG-3′, reverse 5′-AGGAACTGACAGTGGCTTGCT; mouse VEGFR3, forward 5′-CAGGACAGGCGACCATACAG-3′, reverse 5′-TCCACAGGGACAAAGAGGACTA-3′; mouse Podoplanin, forward 5′-TCCGATGAGTTGAGGAGCCA-3′, reverse 5′-AGAAGCAGAAGGCAGGTGTTAGA-3′.

### Flow cytometry

Renal tissue was minced, digested by collagenase, washed with PBS, and finally prepared as a single cell suspension. The cells were then treated at 4 °C for 30 min in the dark with the following antibodies: APC anti-mouse CD11b (BioLegend, USA, 1:100), PE anti-mouse CD86 (BioLegend, USA, 1:50), FITC anti-mouse MHC-II (BioLegend, USA, 1:50), Percp/cy5.5 anti-mouse F4/80 (BioLegend, USA, 1:50), PE-cy7 anti-mouse Podoplanin (BioLegend, USA, 1:50). Flow cytometric analysis was performed using a BD FACSCanto II (BD, USA) and FlowJo software.

### Tube formation assay of BMDMs

Primary BMDMs were harvested and prepared as suspensions. Under precooling conditions, 25 µl of the cell suspension (2 × 10^5^) and 25 µl of reduced growth factor Matrigel (BD, USA) were mixed well, seeded in precooled, 96-well plates, and allowed to gel for 30 min at 37 °C. Then, 200 µl of EBM-2 (Lonza, Switzerland) containing 3% FBS (HyClone, USA) was added and changed every 3 days.

### Statistical analysis

All statistical analyses were conducted using SPSS 12.0 (SPSS, USA). The values are expressed as the mean ± SD. and were used for the statistical analysis with Student’s *t*-test or one-way ANOVA where appropriate followed by Dunnett’s comparison test. The tests were two-sided. Pearson correlation coefficient was used for correlation analysis. The threshold for statistical significance was set at *p* < 0.05. For animal studies, sample size was set to 6. Data collection and analysis are performed by different personnel to implement blinding.

## Supplementary information

S Figure 1

S Figure 2

S Figure 3

Supplementary Figure Legends

## Supplementary information

**Supplementary Information** The online version contains supplementary material available at (10.1038/s41419-020-03385-x).
