# Supplementary figures and images for: Lymphangiogenesis in renal fibrosis arises from macrophages via VEGF-C/VEGFR3-dependent autophagy and polarization
Source: Cell Death Dis. 2021 Jan 21;12(1):109. doi: 10.1038/s41419-020-03385-x (PMC7820012; doi:10.1038/s41419-020-03385-x)

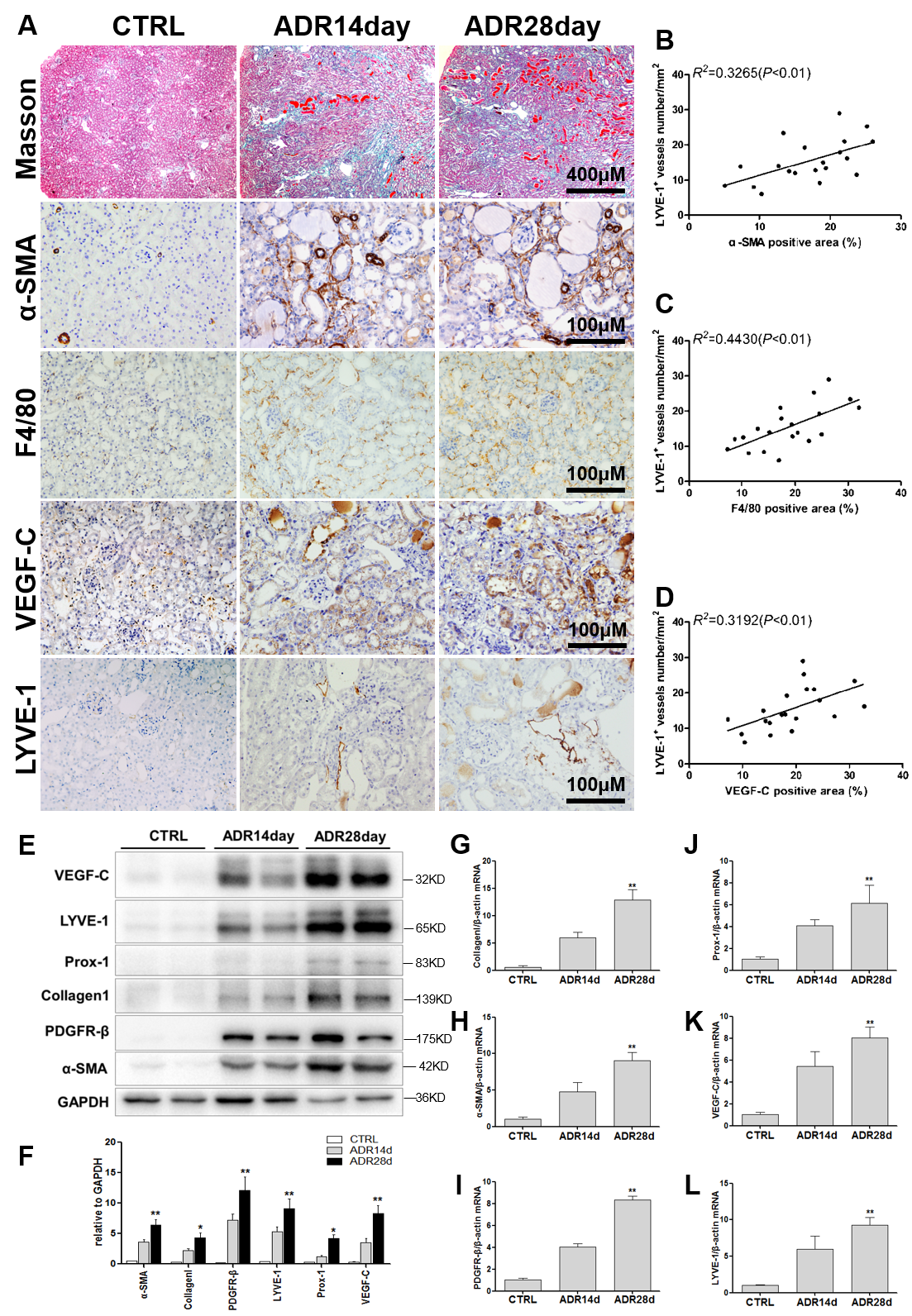

Supplement: Supplementary file 1 — S Figure 1 [file 41419_2020_3385_MOESM1_ESM.tif]

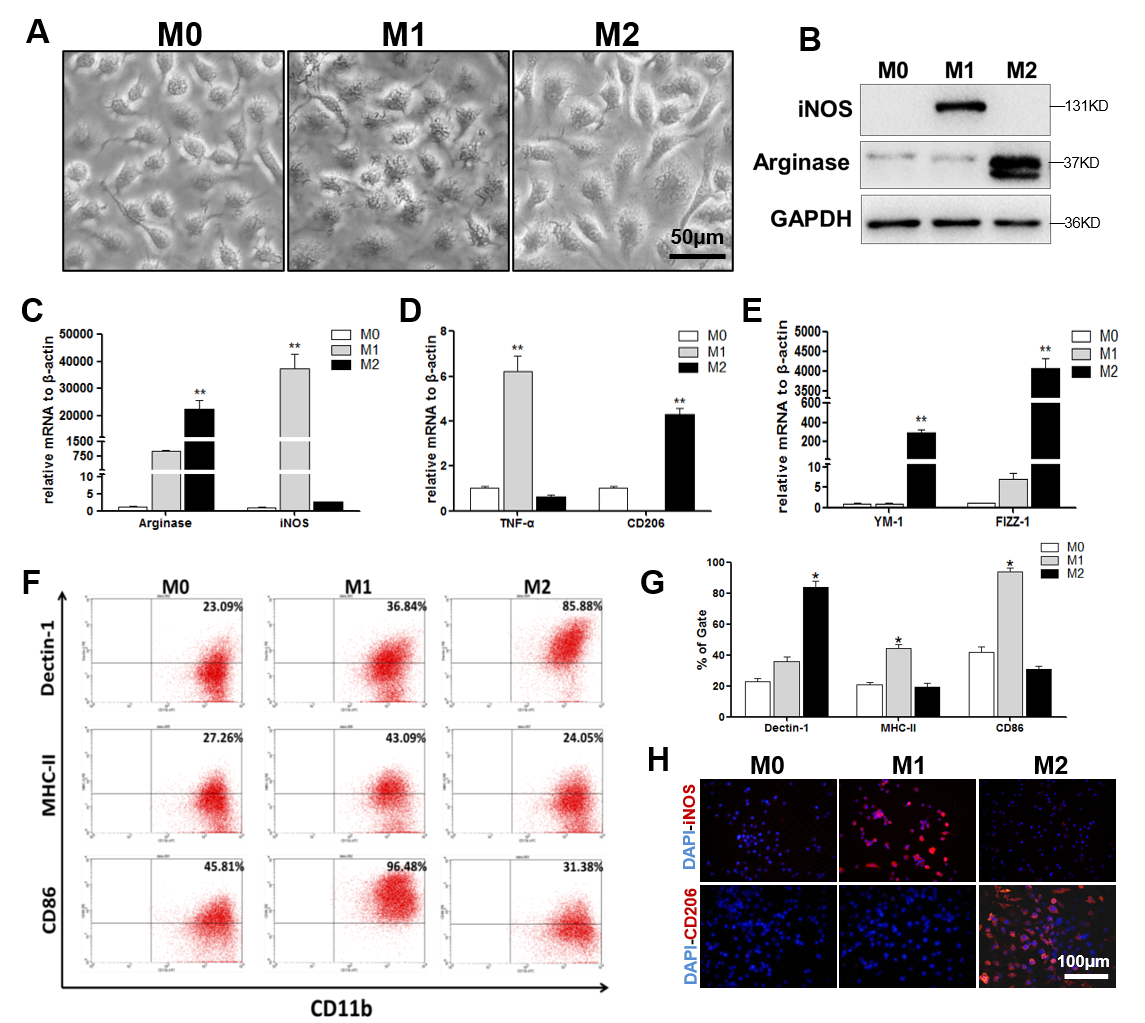

Supplement: Supplementary file 2 — S Figure 2 [file 41419_2020_3385_MOESM2_ESM.tif]

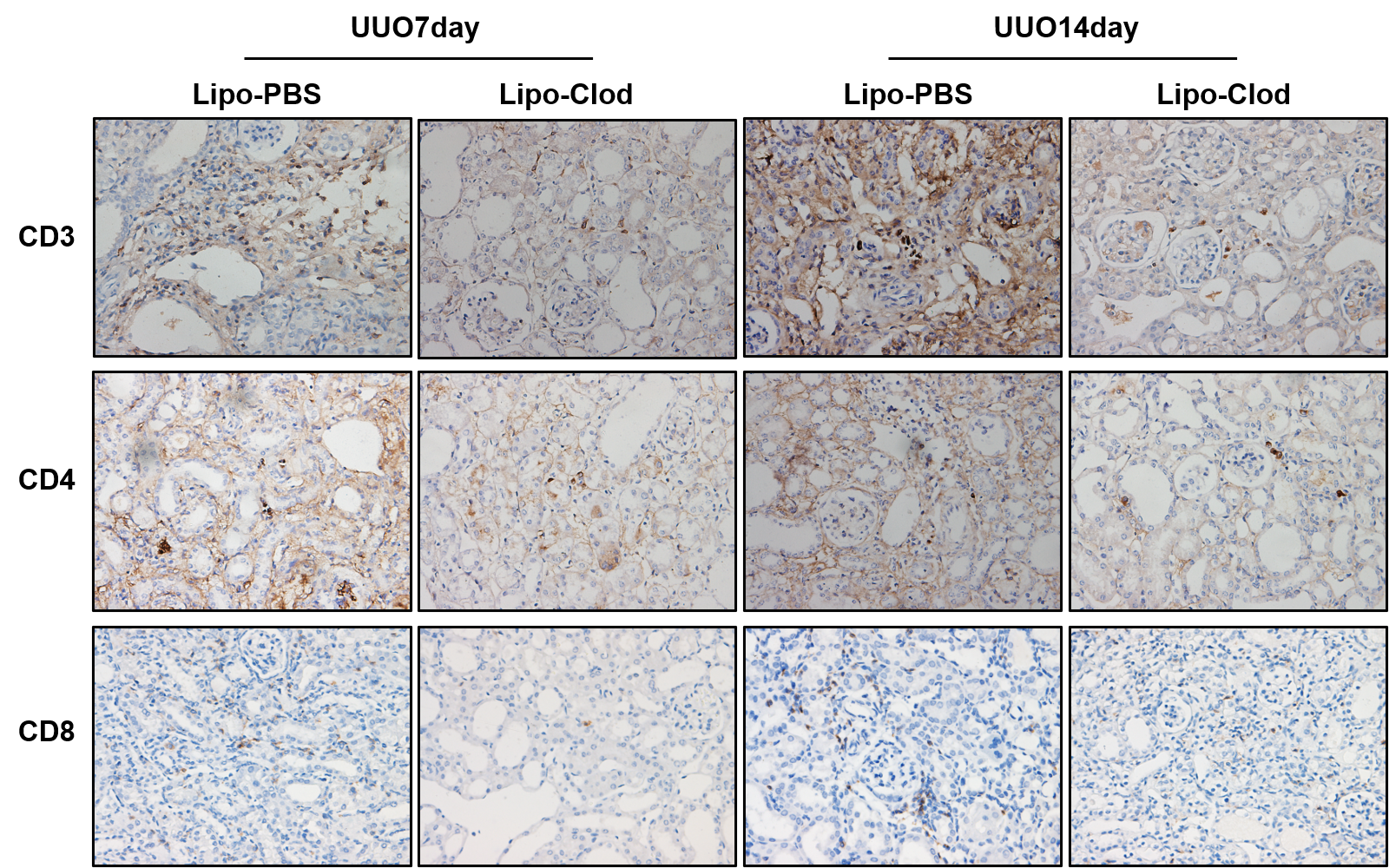

Supplement: Supplementary file 3 — S Figure 3 [file 41419_2020_3385_MOESM3_ESM.tif]
