# Supplementary material for: Lymphangiogenesis in renal fibrosis arises from macrophages via VEGF-C/VEGFR3-dependent autophagy and polarization
Source: Cell Death Dis. 2021 Jan 21;12(1):109. doi: 10.1038/s41419-020-03385-x (PMC7820012; doi:10.1038/s41419-020-03385-x)
Supplement: Supplementary file 4 — Supplementary Figure Legends [file 41419_2020_3385_MOESM4_ESM.docx]

**Figure S1. Renal fibrosis, lymphangiogenesis, macrophage infiltration and VEGF-C expression in ADR mice.**

**(**A) Kidney tissue from saline-treated mice (CTRL) or ADR mice was tested by Masson staining (100×) and immunohistochemical analysis of α-SMA, F4/80, VEGF-C and LYVE-1 (400×). There was a positive correlation between the number of LYVE-1^+^ vessels and the positive areas (%) of α-SMA (B), F4/80 (C) and VEGF-C (D). (E) Protein expression of fibrosis markers (α-SMA, collagen 1, and PDGFR-β) and lymphangiogenesis markers (LYVE-1, Prox-1, and VEGF-C) in CTRL and ADR mouse kidneys were measured by western blotting. (F) Quantitative analysis of the results in E was conducted to show the protein expression of fibrosis markers (α-SMA, collagen 1, and PDGFR-β) and lymphangiogenesis markers (LYVE-1, Prox-1, and VEGF-C) in CTRL and ADR mouse kidneys. *P<0.05, **P<0.01 versus CTRL. (G to L) Relative mRNA expression of collagen 1 (G), α-SMA (H), PDGFR-β (I), Prox-1 (J), VEGF-C (K) and LYVE-1 (L) in CTRL and ADR mouse kidneys was measured by real-time PCR; *p<0.05, **p<0.01 versus CTRL.

**Figure S2. Identification of isolated and activated primary BMDMs.**

(A) Morphology of BMDMs subpopulations. 400×. (B) BMDMs subpopulations were identified by western blot analysis of iNOS and arginase, which are markers for classic activation (M1) and alternative activation (M2), respectively. (C to E) Relative mRNA expression of the macrophage activation markers arginase, iNOS, TNF-α, CD206, YM-1, and FIZZ-1 was measured by real-time PCR in BMDMs subpopulations; *p<0.05, **p<0.01 versus the other two groups. (F and G) Flow cytometry results showed the ratios of CD11b and CD86/MHC-II/Dectin-1 double-positive cells in BMDM subpopulations; G is the statistical graph of the data in F. *p<0.05, **p<0.01 versus the other two groups. (H) BMDM subpopulations were immunofluorescently stained for iNOS and CD206 by immunofluorescence; DAPI is shown in blue. 400×.

**Figure S3. Macrophage depletion by clodronate liposomes downregulated T cells in UUO mice.**

Representative images showing immunohistochemical staining (400×) of CD3^+^ T cells, CD4^+^ T cells and CD8^+^ T cells. Lipo-PBS indicates intraperitoneal injection of PBS liposomes as a control, and Lipo-Clod indicates intraperitoneal injection of clodronate liposomes.
